# Diagnostic Performance of Coccidioidomycosis PCR Testing in Lung Nodules: A Retrospective Study in Central California

**DOI:** 10.3390/jof11110814

**Published:** 2025-11-16

**Authors:** Faisal Nasrawi, Mohamed A. Fayed, Michael W. Peterson

**Affiliations:** 1 Pulmonary Critical Care Division, UCSF Fresno, Fresno, CA 93701, USA; 2 UC Merced Health Sciences Research Institute, Merced, CA 95343, USA

**Keywords:** Coccidioidomycosis, *Coccidioides*, diagnostic yield, endemic fungal infection, fungal culture, histopathology, lung nodules, molecular diagnostics, PCR, sensitivity, pulmonary mycosis, valley fever

## Abstract

Background: Coccidioidomycosis is an endemic fungal infection in the southwestern United States that can present as solitary lung nodules, mimicking malignancy on imaging. Molecular testing, such as PCR, offers rapid diagnosis but its performance in this clinical setting remains unclear. Methods: We conducted a retrospective analysis of patients evaluated for lung nodules at a tertiary care community-based lung nodule clinic in Central California between 2011 and 2025. *Coccidioides* PCR in patients with proven or probable Coccidioidomycosis was compared to those with biopsy-proven lung cancer. Diagnostic yield of *Coccidioides* PCR was assessed across biopsy methods and benchmarked against histology and fungal cultures. Results: Among 122 patients with Coccidioidomycosis, PCR demonstrated low sensitivity (range: 20–41%) but high specificity (100%) across all biopsy modalities. Histology and fungal cultures outperformed PCR, detecting additional cases missed by molecular testing. Notably, 9 PCR-negative cases were confirmed on histology, and PCR was only positive in 71.4% of culture-confirmed cases. Conclusion: *Coccidioides* PCR testing has high specificity but limited sensitivity for diagnosing lung nodules in endemic regions, limiting its utility as a single test. Histology and fungal culture remain essential. Selective use of PCR may enhance diagnostic efficiency and reduce unnecessary costs in regions burdened by Coccidioidomycosis.

## 1. Introduction

Coccidioidomycosis (due to *Coccidioides* spp.) is a fungal infection endemic to arid regions of the southwestern United States, including California’s Central Valley, Arizona, Nevada, and parts of Texas and New Mexico. The infection is acquired through inhalation of airborne fungal spores, which originate from disturbed soil in endemic areas, particularly after dust storms or construction activities [1]. The clinical spectrum of disease varies widely, ranging from asymptomatic infection to severe disseminated disease. While most infections are self-limited, approximately 40% of infected individuals develop symptomatic pulmonary disease, which can mimic bacterial or viral pneumonia [2]. In both symptomatic and asymptomatic pulmonary infections, a nodule may form that can mimic lung cancer and is often discovered incidentally on chest imaging performed for other reasons [3,4]. These nodules, which may be solitary or multiple, can be indistinguishable from malignancy based on radiographic features alone [5]. Consequently, differentiating benign and malignant nodules is critical because it influences patient management.

Molecular diagnostic tools such as PCR assays for *Coccidioides* DNA offer a potential solution for improving diagnostic accuracy. The original Cocci PCR assay described by Binnicker et al. demonstrated excellent performance, with 100% sensitivity and 98.4% specificity for respiratory specimens under controlled laboratory conditions [6]. However, the study did not specify whether the respiratory specimens were collected from patients with acute infection or whether they included samples from patients with lung nodules or asymptomatic disease. Similarly, the GeneSTAT assay demonstrated 100% sensitivity for bronchoalveolar lavage (BAL) and bronchial wash specimens, with a specificity range of 93.8–100% [7], but again it was unclear if the tested samples came from patients with active infection or from pulmonary nodules.

Despite these promising results, variability in PCR performance has been noted in real-world settings, particularly across different specimen types and patient populations. For example, the BD MAX system demonstrated an overall sensitivity of 74% in a mixed cohort of respiratory and tissue specimens, but sensitivity varied depending on the sample type. BAL had 91% sensitivity in patients with acute pneumonia, and sputum was 94% sensitive. However, the BD MAX system demonstrated much lower sensitivity for tissue samples [8]. It was unclear whether this was due to small sample size or failure to successfully obtain tissue from the lesion.

The purpose of our current study is to evaluate the diagnostic performance of *Coccidioides* PCR using the BD MAX system in a larger cohort of patients with lung nodules with histologic or smear/culture confirmation. We evaluated patients who underwent biopsy and were diagnosed with nodules due to Coccidioidomycosis or lung cancer seen in our lung nodule clinic between 2011 and 2024 and who had *Coccidioides* PCR testing.

## 2. Clinical Challenge: A Representative Case

**Figure 1:**
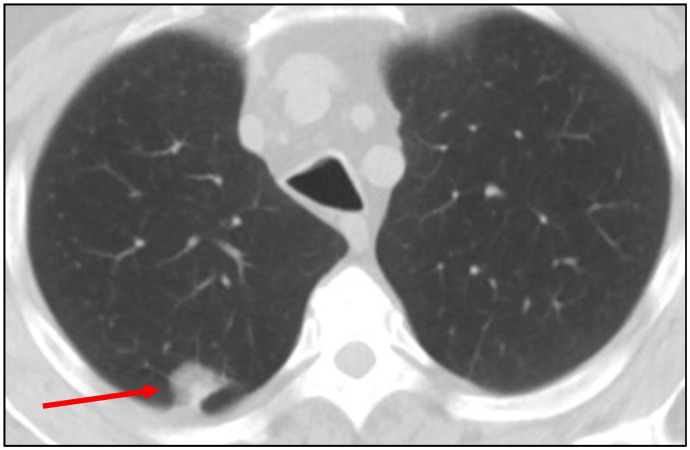
Representative CT image showing a spiculated right upper lobe nodule suggestive of malignancy, later diagnosed as Coccidioidomycosis.

To highlight the difficulty in differentiating lung nodules due to Coccidioidomycosis from those due to lung cancer, we present this representative case. A 69-year-old male with a 60-pack-year history of smoking presented for evaluation of an incidentally detected pulmonary nodule on chest CT. The 2.4 cm nodule was in the right upper lobe, with no calcifications or satellite lesions, raising high suspicion for primary lung malignancy (Figure 1).

Given the radiographic features and clinical risk, the patient underwent a CT-guided transthoracic biopsy. Pathology showed reactive lung and necrotic tissue with round spherules and focal chronic inflammation, suspicious for Coccidiomycosis. Grocott methenamine silver (GMS) stain was positive for fungal organisms, morphologically compatible with *Coccidioides*, confirming Coccidioidomycosis as the diagnosis.

**Figure 2:**
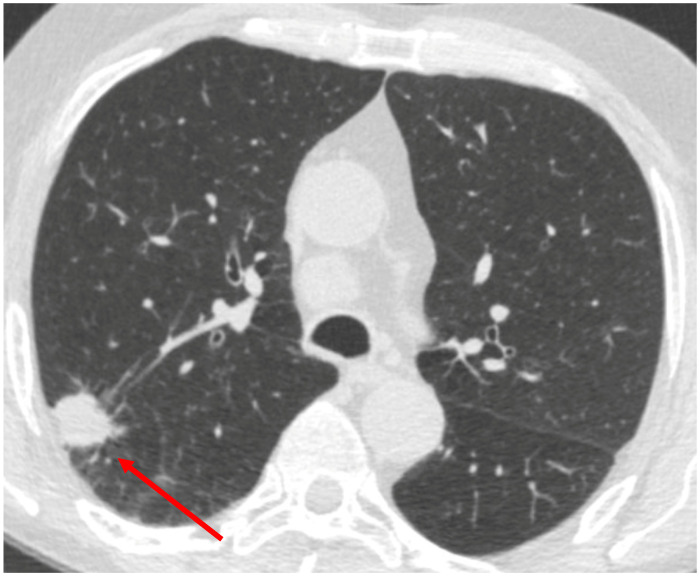
Representative CT image showing a spiculated right upper lobe nodule suggestive of malignancy, ultimately diagnosed with non-small cell lung carcinoma.

To further underscore the diagnostic challenge, we also include a representative CT scan from a patient who ultimately was diagnosed with primary non-small cell lung carcinoma (Figure 2). This lesion shared similar radiographic characteristics with the coccidioidal nodule, including irregular borders, absence of calcifications, and concerning size; features that in both cases raise strong suspicion of malignancy. The striking overlap in imaging appearance highlights how coccidioidal nodules can closely mimic malignant ones, reinforcing the need for tissue sampling and multimodal diagnostic approaches in endemic regions.

## 3. Materials and Methods

### 3.1. Study Design and Patient Selection

We conducted this retrospective study at UCSF Fresno and Community Medical Centers Lung Nodule Program, a multidisciplinary lung cancer evaluation center in Fresno, CA, affiliated with the UCSF Fresno regional campus. The program specializes in the rapid diagnosis and management of pulmonary nodules, with a primary focus on detecting and staging lung cancer. It is staffed by UCSF Fresno pulmonary faculty, with expertise in lung cancer as well as Coccidioidomycosis.

**Figure 3:**
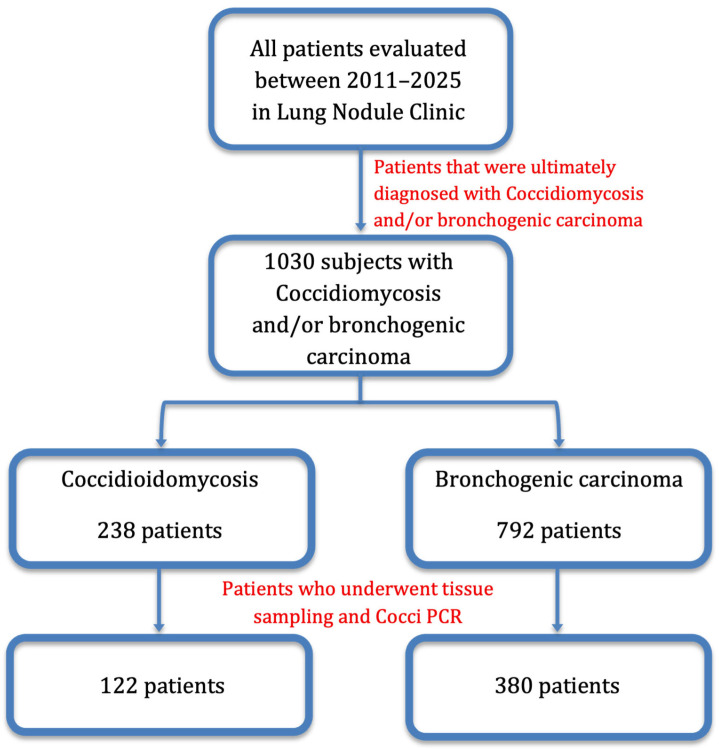
Patient Selection Flowchart: Derivation of Coccidioidomycosis and Malignancy Cohorts.

Patients evaluated in the Lung Nodule Program undergo a structured diagnostic workup, which includes standardized clinical risk assessment (smoking history including pack years, personal history of cancer, family history of lung cancer, occupational exposures, and assessment for chronic lung disease), imaging, molecular testing, and biopsy procedures. Depending on the nodule location and risk assessment, patients undergo biopsy by transthoracic needle biopsy under CT guidance, bronchoscopy, navigational bronchoscopy (both robotic and non-robotic), or endobronchial biopsy. We analyzed the diagnostic performance of *Coccidioides* PCR testing for lung nodules using histology and fungal cultures as gold standards. Patient data was collected from 2011 to 2025. The case group consisted of patients with evidence of *Coccidioides* infection, while the control group were patients with lung nodules due to bronchogenic carcinoma. In total, 238 patients had proven or probable coccidioidomycosis, and 792 patients had biopsy-proven lung cancer. Among the Coccidioidomycosis group, 122 patients had *Coccidioides* PCR performed, and among the control group, 380 patients underwent *Coccidioides* PCR (Figure 3). The decision to order *Coccidioides* PCR was made at the discretion of the treating physician.

Patients were classified as “proven” or “probable” coccidioidomycosis based on EORTC/MSGERC criteria [9]. All patients resided in a geographic area endemic to *Coccidioides*. Proven Coccidioidomycosis was defined by direct microbiological or histopathological evidence of infection, including identification of *Coccidioides* spherules on histologic examination and/or positive fungal culture for *Coccidioides* spp. Probable Coccidioidomycosis was defined by clinical, radiographic, histopathological, and/or serological findings consistent with Coccidioidomycosis infection but lacking direct microbiological confirmation. These findings included positive *Coccidioides* serology (IgM and/or IgG) with compatible clinical presentation and/or granulomatous inflammation on pathology, in combination with clinical and radiographic findings suggestive of Coccidioidomycosis, and with stability or improvement in nodule(s) with or without antifungal therapy.

Institutional review board (IRB) approval was obtained for data collection and analysis (IRB #2011075).

### 3.2. Specimens

Biopsy material included both formalin-fixed paraffin-embedded (FFPE) tissue blocks and fresh tissue specimens. For FFPE samples, 5–10 μm sections were prepared using sterile, DNA-free blades. For fresh tissue, specimens were handled under biosafety level-3 precautions until inactivation. All samples underwent DNA extraction and amplification on the BD MAX™ System (Becton Dickinson, Sparks, MD, USA) using the BioGX fungal panel reagents, following manufacturer instructions and protocols optimized for FFPE material.

### 3.3. Histopathology

**Figure 4:**
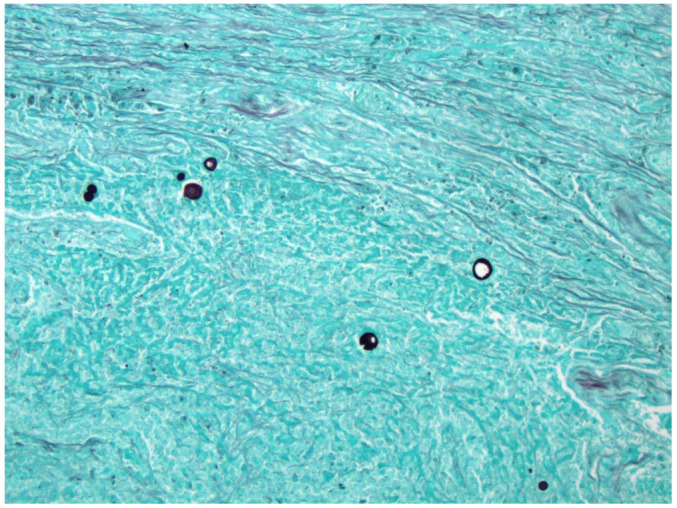
GMS stain showing *Coccidioides* spherules.

All biopsies were reviewed by board-certified pathologists. Hematoxylin and eosin (H&E) stains were routinely performed, and special stains including Grocott methenamine silver (GMS) and Periodic acid–Schiff (PAS) were used when fungal organisms were suspected. Representative histologic images of spherules are shown in Figure 4.

### 3.4. PCR Testing

Molecular detection of *Coccidioides* DNA was performed using a real-time PCR assay on the BD Max system (Becton Dickinson, Franklin Lakes, NJ, USA) as previously described. The assay targeted the internal transcribed spacer 2 (ITS2) region of ribosomal DNA, using primers validated for Coccidioides spp. as described by Binnicker et al. [6,10], The ITS2 region, though conserved among fungi, contains sufficient sequence divergence for species-level identification. All assays included internal amplification controls to confirm DNA integrity and rule out inhibition. Each BD Max ExK DNA-1 reagent strip contained master mix reagents (BioGX), internal amplification control, primers, and fluorescent probes. Reactions were run under the following cycling conditions: denaturation, annealing/extension, and fluorescence acquisition for 45 cycles. Amplification data were analyzed using the BD Max automated molecular platform.

A result was interpreted as positive if the amplification curve crossed the threshold before cycle 44; negative if no amplification occurred by cycle 45. Results between cycles 42–44 were interpreted cautiously and required curve morphology consistent with true amplification. A separate melting curve analysis was not performed, as amplification curve morphology and internal control thresholds were used for validation per BD MAX protocol.

### 3.5. Controls and Quality Assurance

Each run included an internal control (IC) incorporated into every cartridge to confirm successful amplification and absence of PCR inhibition. The internal amplification control (IAC) included in every cartridge confirmed successful extraction and amplification, ensuring assay reliability. An external positive control consisted of heat-killed *Coccidioides immitis* culture diluted into BAL fluid, processed alongside clinical specimens. A no-template negative control (sterile water) was included in each batch. Analytical specificity was verified against nucleic acids from related fungal pathogens during assay validation. Ongoing laboratory quality assurance included proficiency testing every six months.

### 3.6. Statistical Analysis

Diagnostic performance metrics, including sensitivity, specificity, positive predictive value (PPV), negative predictive value (NPV), and accuracy were calculated for PCR, along with their respective confidence intervals (CIs), using the online tool MedCalc [11].

## 4. Results

### Diagnostic Performance

Of the 238 patients with Coccidioidomycosis, 122 had biopsies or washings that included PCR (Figure 3). These included 65 CT-guided biopsies, 22 standard bronchoscopies, 15 navigational bronchoscopies, and 20 endobronchial ultrasound-guided (EBUS) lymph node biopsies. A control group of 380 patients with biopsy-confirmed malignancy who also had *Coccidioides* PCR testing was analyzed for comparison. This group included 231 CT-guided biopsies, 53 standard bronchoscopies, 43 navigational bronchoscopies, and 53 EBUS lymph node biopsies.

Among the 122 patients with coccidioidomycosis who underwent PCR testing, 31 were PCR positive and 91 were PCR negative (Table 1). Across biopsy modalities, the highest percentage positive was in bronchoscopy (40.9%) followed by EBUS at 30% and both navigational bronchoscopy and CT biopsy at 20%. In all modalities, the majority of Coccidioidomycosis cases yielded negative PCR results. In the 380 malignant nodule controls that underwent PCR testing, all samples tested negative with no false positives.

The diagnostic performance of Coccidioides PCR testing across various invasive procedures is summarized in Table 2. Sensitivity was notably low across all methods, with the highest observed in bronchoscopy samples (40.91%). Specificity remained consistently high at 100% across all procedures.

To better understand the diagnostic value of PCR, we also analyzed the performance of histology and fungal culture. Among the 122 biopsied patients with proven or probable Coccidioidomycosis, histology demonstrated the highest overall diagnostic yield at 47.5%, clearly outperforming both fungal culture (23.8%) and PCR (25.4%). The low diagnostic yield of PCR aligns with its poor sensitivity observed elsewhere in our analysis. Among the 122 patients from the coccidiomycosis group, PCR was positive in 31 cases, while histology identified 98 and culture 37. PCR results were concordant with histology in only 16 patients and with culture in 14. Importantly, PCR was positive in the absence of positive culture or histology in only two patients. In one case, histology showed “yeast” forms that may have represented single endospores, and in the other, mycelial organisms were observed which can rarely be seen in *Coccidioides* infections [12]. By contrast, PCR was negative in 82 patients who were histology-positive and 23 who were culture-positive for coccidioidomycosis. PCR contributes little unique diagnostic value in the setting of pulmonary nodules. No false-positive PCR results were observed, confirming its high specificity.

PCR was negative in approximately three out of every four cases of proven or probable coccidioidomycosis. In practice, a negative PCR is not reliable for ruling out disease. With a pre-test probability of 24.3% as seen in our sample, the negative likelihood ratio (0.75) reduces the post-test probability to ~19%, which is insufficient to exclude infection. By contrast, given the 100% specificity observed in our control group, a positive PCR result is highly confirmatory.

## 5. Discussion

Increasing numbers of chest CT scans are being performed, and lung nodules are a common finding on chest CT scans [13]. Because lung nodules can represent early lung cancer, it is important that they be evaluated in a timely and accurate manner. In Coccidioidomycosis endemic areas, the fungus can cause 25–50% of the lung nodules seen [4,14], and our data are consistent with that; 23% of our nodules are pathologically attributable to Coccidioidomycosis. This strikingly high proportion underscores the significant burden of Coccidioidomycosis nodules masquerading as potential malignancies in an endemic area. In addition to the anxiety and potential complications associated with evaluating lung nodules, Coccidioidomycosis imposes a substantial financial burden in endemic regions. A cost analysis by Grizzle et al. estimated that the lifetime economic burden of 10,359 incident cases of Coccidioidomycosis in Arizona was approximately $736 million, including $671 million in direct medical costs and $65 million in indirect costs, with an average direct cost of $64,800 per patient [15]. Similarly, Wilson et al. reported that 7466 incident cases in California in 2017 were associated with $429 million in direct and $271 million in indirect lifetime costs, totaling nearly $700 million, with an average per-patient direct cost of $57,413 [16]. Pulmonary nodules due to Coccidioidomycosis were the second most expensive clinical presentation of *Coccidioides* infection. This is due to the frequent use of advanced imaging, invasive biopsies, and specialist referrals [15,16]. It is thus incumbent on us to provide the most cost-effective care we can for these patients.

Because fungal cultures may take weeks to grow, and histology may require 5–7 days to complete, molecular studies including PCR hold the promise for fast and accurate diagnosis. While prior studies demonstrated high sensitivity and specificity of PCR in respiratory specimens, none of the studies focused specifically on lung nodules.

Earlier studies, such as Binnicker et al., reported PCR sensitivity exceeding 85% in respiratory specimens. While those publications did not clearly specify the clinical context of the patients from whom samples were obtained, they may have reflected acute symptomatic infections. By contrast, our study was conducted in an outpatient lung nodule clinic, where most patients were asymptomatic and evaluated for indolent pulmonary nodules. This difference in patient population is a key factor in understanding why PCR may perform differently in our cohort.

It is also important to note that the PCR platform used in this study has previously demonstrated high sensitivity at our center for acute infections, consistent with results reported by Binnicker. In prior publications from our institution [8], PCR performance in acute or disseminated cases was strong, confirming that the assay itself is robust. This supports the interpretation that the poor sensitivity observed in this study is due to the patient population and clinical setting rather than deficiencies in the PCR method itself.

Our study focuses on lung nodules with tissue confirmation that PCR is being performed on appropriate pathological specimens. Our findings do align with Dizon et al. who reported a sensitivity of 74% across diverse specimen types but only 44% in tissue samples for lung nodules [8]. However, the Dizon study had few lung nodules and did not have histologic confirmation. Our findings address both of these shortfalls and support the need to re-evaluate the role of PCR in the diagnostic workup of pulmonary nodules, given its low sensitivity and high cost.

Not all patients in the Coccidioidomycosis group underwent PCR; 122/238 (51%) had testing, reflecting provider discretion at the time of biopsy. This nuance is important when interpreting sensitivity values, as they reflect a subset of patients rather than the entire case cohort. Given the assay’s low sensitivity in this setting (25.4%) and an NPV of 80.7%, a negative PCR result does not exclude coccidioidomycosis in patients evaluated for lung nodules. By contrast, the 100% specificity demonstrates that a positive PCR is highly specific and can be considered confirmatory in the appropriate clinical context.

It is not clear why *Coccidioides* PCR performs so differently in lung nodules compared to other clinical forms of the disease. Cases where PCR was negative, but histology or culture confirmed Coccidioidomycosis, highlight potential technical or biological limitations. For instance, the localized fungal burden in nodules may fall below the assay detection threshold, and DNA degradation during sample processing or the presence of PCR inhibitors could contribute to false negatives.

These limitations parallel observations in tuberculosis (TB) diagnostics, where PCR sensitivity is lower for latent infections than for active TB. Such patterns emphasize the importance of tailoring diagnostic tools to the clinical context [17,18].

In our cohort, PCR testing was performed exclusively for Coccidioides spp., consistent with regional epidemiology in California’s Central Valley, where Coccidioidomycosis is the predominant fungal cause of pulmonary nodules. While fungal culture occasionally identified other organisms, such as Aspergillus, molecular testing for non-endemic fungi was not conducted. Whether PCR assays for these pathogens would show similar diagnostic performance in tissue samples remains uncertain, and routine use of such assays in non-endemic settings is unlikely to offer substantial clinical benefit.

In this cohort, serology was part of the definition of probable coccidioidomycosis; however, individual serology results were not systematically tracked for analysis, and therefore we cannot correlate them directly with PCR results. Based on our clinical experience, serology remains a helpful diagnostic tool, and future studies integrating serology with molecular and histopathologic testing may better define its role in patients presenting with pulmonary nodules.

While many consider Coccidioidomycosis a “rare” disease because of its limited geographic distribution, more than 35 million people live in the endemic regions in the United States, and many additional people travel to these areas each year. In addition, recent studies show that the number of cases is growing significantly in endemic regions, and the disease is massively underreported with an estimated annual disease burden for 2019 of 206,000 to 360,000 symptomatic cases [19]. Furthermore, climate modeling predicts further expansion of the endemic region into most of the western United States by 2060 [20,21].

Although our findings demonstrate excellent specificity with no false-positive results, it is important to recognize that PCR detects fungal DNA regardless of organism viability. Residual DNA from prior infection or environmental contamination could theoretically yield positive results in the absence of active disease. Moreover, a positive PCR result does not necessarily indicate that antifungal treatment is warranted. Active coccidioidomycosis is a clinical syndrome, and management decisions should be guided by a patient’s symptoms, radiographic evolution, risk factors, and overall clinical context. Initiating antifungal therapy based solely on a positive PCR should be approached with caution and involve clinicians experienced in the diagnosis and management of Coccidioidomycosis. This is particularly true in asymptomatic patients with incidentally discovered nodules. Thoughtful multidisciplinary evaluation remains essential to avoid unnecessary treatment in patients with resolved or inactive infection. In our experience, asymptomatic patients with incidentally detected coccidioidal nodules are typically observed without antifungal therapy unless there is evidence of active disease progression.

### 5.1. Future Directions

To optimize the use of *Coccidioides* PCR in endemic regions, we recommend:

1. Selective Application: Limit routine PCR testing to patients with clinical or radiological signs of active infection to improve cost-effectiveness.
2. Protocol Optimization: Investigate methods to enhance DNA extraction and amplification in low-burden infections.
3. Integrated Risk Models: Incorporate PCR findings into a comprehensive risk calculator that accounts for endemic factors, patient history, and radiologic features.
4. Combined diagnostics: Future studies should evaluate PCR in conjunction with serologic assays and fungal biomarkers (e.g., galactomannan, β-D-glucan) to determine the added value of multimodal strategies.
5. Evaluation of alternative primer sets targeting multicopy genes or species-specific loci may further improve assay sensitivity in low-burden tissue samples.

### 5.2. Study Limitations

Our retrospective design and single-center focus may limit generalizability. Additionally, variations in sample handling and processing over the study period could have influenced PCR performance. Future multicenter studies with standardized protocols are needed to validate our findings.

### 5.3. Study Strengths

This study has several strengths that enhance the reliability of its findings. The large sample size provides a robust dataset for assessing the diagnostic accuracy of *Coccidioides* PCR in lung nodules, making this one of the largest single-institution studies on this topic. Additionally, the study population was derived from a dedicated lung nodule clinic, ensuring that all cases underwent a structured diagnostic approach that provides the best evidence-based real-world clinical practice. The use of histology and fungal culture as gold standards strengthens the validity of the conclusions. Lastly, the study was conducted in a highly endemic region, where differentiating between Coccidioidomycosis and malignancy remains a significant clinical challenge, further underscoring the importance of these findings.

## 6. Conclusions

*Coccidioides* PCR has high specificity but low sensitivity when used for lung nodules in an endemic region, limiting its role as a standalone diagnostic tool. Histology and fungal culture remain essential for diagnosis because they demonstrated higher sensitivity than PCR. Negative PCR results should not exclude Coccidioidomycosis, because false negatives are common in this setting.

Given these findings, *Coccidioides* PCR should be used selectively, particularly in cases where culture and histology are unavailable or impractical. Future studies should focus on optimizing PCR protocols for lung nodules, evaluating alternative molecular markers, and integrating PCR with imaging and serology into a comprehensive diagnostic approach for pulmonary nodules in Coccidioidomycosis-endemic regions.

## Figures and Tables

**Table 1:** Performance of *Coccidioides* PCR by Biopsy Modality.

	CT Biopsy	Bronchoscopy	Navigational	EBUS	Total/Combined
True positive	13	9	3	6	31
False Negative	52	13	12	14	91
Percent Positive	20%	40.9%	20%	30%	25.4%
True Negative	231	53	43	53	380
False positive	0	0	0	0	0

**Table 2:** Diagnostic Performance Metrics by Procedure.

Procedure	Sensitivity (%) (CI)	Specificity (%) (CI)	PPV (%) (CI)	NPV (%) (CI)	Accuracy
CT-guided biopsy	20.00 (11.10–31.77)	100.00 (98.42–100.00)	100.00 (75.29–100.00)	81.63 (79.73–83.38)	82.43 (77.61–86.59)
Bronchoscopy	40.91 (20.71–63.65)	100.00 (93.28–100.00)	100.00 (66.37–100.00)	80.30 (74.22–85.23)	82.67 (72.19–90.43)
Navigational bronchoscopy	20.00 (4.33–48.09)	100.00 (91.78–100.00)	100.00 (29.24–100.00)	78.18 (73.56–82.19)	79.31 (66.65–88.83)
EBUS biopsy	30.00 (11.89–54.28)	100.00 (93.28–100.00)	100.00 (54.07–100.00)	79.10 (73.97–83.45)	80.82 (69.92–89.10)
Combined	25.41 (17.96–34.09)	100.00 (99.03–100.00)	100.00 (88.78–100.00)	80.68 (79.01–82.24)	81.87 (78.22–85.15)

## Data Availability

The original contributions presented in this study are included in the article. Further inquiries can be directed to the corresponding author.
